# Physical Properties, α-Glucosidase Inhibitory Activity, and Digestive Stability of Four Purple Corn Cob Anthocyanin Complexes

**DOI:** 10.3390/foods11223665

**Published:** 2022-11-16

**Authors:** Jialin Dai, Yanye Ruan, Ying Feng, Bin Li

**Affiliations:** 1 Food College, Shenyang Agricultural University, Shenyang 110866, China; 2 College of Bioscience and Biotechnology, Shenyang Agricultural University, Shenyang 110866, China

**Keywords:** purple corn anthocyanin, physicochemical properties, bioactivity, structural characterization, in vitro gastrointestinal digestion

## Abstract

In this study, pectin (PC), whey protein isolate (WPI), and chitosan (CS) were combined with purple corn cob anthocyanins (PCCA). Four complexes, PC−PCCA, WPI−PCCA, WPI−PC−PCCA, and CS−PC−PCCA were prepared to evaluate the improvement in the α-glucosidase inhibitory activity and digestive stability of PCCA. The encapsulation efficiency (EE), particle size, physical properties, and mode of action of the synthesized PCCA complexes were evaluated. Among them, CS−PC−PCCA had the highest EE (48.13 ± 2.73%) except for WPI−PC−PCCA; furthermore, it had a medium size (200−300 nm), the lowest hygroscopicity (10.23 ± 0.28%), lowest solubility (10.57 ± 1.26%), and highest zeta potential (28.20 ± 1.14). CS−PC−PCCA was multigranular and irregular in shape; x-ray diffraction showed that it was amorphous; and Fourier transform infrared spectroscopy confirmed that it was joined with PCCA through hydrogen bonds and electrostatic interactions. Compared with PCCA, the four complexes showed a higher α-glucosidase inhibition activity and digestive stability, except for WPI−PC−PCCA. Furthermore, CS−PC−PCCA exhibited the best α-glucosidase inhibition and simulated digestion stability.

## 1. Introduction

Anthocyanins are bioactive phytochemicals that have a variety of colours that are affected by different pH values and are widely found in black beans, purple corn, purple sweet potato, etc. [1]. Purple corn cob (PCC) is rich in anthocyanins [2]. Studies have found that purple corn cob anthocyanins (PCCA) are widely studied because of their various physiological activities, including their role in reducing blood glucose, scavenging free radicals, and inhibiting obesity [3,4]. However, the stability of anthocyanins is easily affected by pH, light, temperature, oxygen, and the gastrointestinal environment, resulting in a lower bioaccessibility and a limited application [5,6,7].

Studies have shown that encapsulation technologies, such as microencapsulation, liposomes, lotion encapsulation, and nanoparticles, can stabilize their bioactivity [7]. In recent years, proteins and polysaccharides have been widely used as combination steady-state materials for anthocyanins [5]. Whey protein isolate (WPI), pectin (PC), and chitosan (CS) are such protein and polysaccharides. WPI is a protein that contains a large amount of α-globulin, which has a high hydrophilicity and various functional properties and has been widely used as a wall material to protect bioactive components [8]. PC is a natural anionic polysaccharide [9]. Since it is very stable in the gastrointestinal tract, it is used as a drug carrier in the pharmaceutical field [10,11]. CS is a natural nontoxic cationic binary heteropolysaccharide with a good biocompatibility, and it has been widely used as an embedding material [12]. However, CS easily dissolves at a low pH, limiting its application in the delivery of bioactive substances. Therefore, the combination of positively charged CS and negatively charged wall materials for embedding has been extensively studied [10].

Khalifa et al. [13] found that WPI can form a stable complex with mulberry anthocyanins through hydrophobic interactions, thereby improving the colour intensity and stability of anthocyanins. Koh et al. [14] found that a complex of blueberry pectin and anthocyanins improves the colour stability and gastrointestinal digestive stability of anthocyanins, which are combined through hydrogen bonds and electrostatic interactions. Zhao et al. [10] found that a bilberry anthocyanin nanocomposite prepared using CS and PC had a good photostability and could be released slowly in the gastrointestinal tract. In addition, anthocyanin combined with β-lactoglobulin and CS derivatives was found to have a stronger heat and light resistance, an in vitro digestive stability, and a slower release than before it was combined [15]. Although Ren and Giusti [16] found that whey protein can protect PCCA against destruction by ascorbic acid in beverages and increase its half-life, there have been few reports on the complexation of PCCA with such proteins or polysaccharides. In addition, CS is easily dissociated and WPI is easily denatured and dissociated in acidic conditions of gastric juice, thus reducing its stability [15,17], while PC is very stable in the gastrointestinal environment [9], therefore, it is of research significance to combine these materials to stabilize anthocyanins and screen the complex with a better stability.

In this study, PC, WPI, and CS were combined with PCCA to stabilize PCCA. CS and PC have good delivery effects as wall materials to prepare polysaccharide, protein, and protein−polysaccharide anthocyanin steady-state complexes. The physical properties, the α-glucosidase inhibitory activity, and the in vitro digestive stability of the four encapsulated complexes, PC−PCCA, WPI−PCCA, WPI−PC−PCCA, and CS−PC−PCCA, were compared. Additionally, their combined action was explored. The results of this study can help accelerate the development of related products and further the use of PCCA in the industry.

## 2. Materials and Methods

### 2.1. Preparation of PCCA

Dried PCC (Heinuo 5) were crushed using a multifunctional grinder (Yongkang hongtaiyang electromechanical Co., Ltd., Yongkang, China) and filtered with a 40-mesh sieve. The obtained powder was placed in absolute ethanol and 0.2 mol/L of citric acid solution (7:3) for the PCCA extraction under conditions of the solid−liquid ratio of 1:63 (g/mL) and an ultrasonic temperature and power of 58 °C and 240 W, respectively, for 41 min. The extract was evaporated in a vacuum at 40 °C and freeze-dried. The dried extract was dissolved in a pH 4.0 citric acid buffer solution at a concentration of 0.03 mg/mL. D-101 resins (Non-polar, Solebao Technology Co., Ltd., Beijing, China) were wet-packed into a glass column (1.0 cm × 30 cm) with a resin bed of 200 mL. According to the adsorption capacity of the resin, eleven bed volumes (BVs) of the above solution were loaded onto D-101 resins at 1 min/mL. The loaded resin was washed with distilled water and 10% ethanol (pH = 2) to remove the impurities and it was subsequently eluted with 30% ethanol (pH = 2) to collect the eluent, which was freeze-dried (LG0.2; Xinyang quick freezing equipment manufacturing Co., Ltd., Shenyang, China) to obtain PCCA.

### 2.2. Preparation of PCCA Complex

With a slight modification, PC−PCCA, WPI−PCCA, WPI−PC−PCCA, and CS−PC−PCCA were prepared, according to the description of Koh et al. [14], Zang et al. [8], Oancea et al. [18], and Zhao et al. [10], respectively.

PC−PCCA: the PC solution (6 mg/mL) and PCCA solution (4 mg/mL) were prepared using a glacial acetic acid buffer (25 mM) at a pH of 4.0, mixed in equal volumes, and allowed to stand for 18 h. After centrifuging (8000× *g*, 30 min), the sediment was collected and freeze-dried to obtain a complex.

WPI−PCCA: the WPI solution (1.5 mg/mL) was prepared with 0.1 M of citric acid buffer solution (pH 6.6) and was rehydrated overnight at 4 °C. The PCCA solution (2.5 mg/mL) was prepared using a pH 3.0 citric acid buffer solution with the same concentration, and both solutions were mixed in equal proportions. After centrifuging (8000× *g*, 30 min), the sediment was collected and freeze-dried to obtain a complex.

WPI−PC−PCCA: the WPI solution (5%, *w*/*w*) and PC solution (2%, *w*/*w*) were prepared with deionized water, rehydrated overnight at 4 °C, subsequently mixed in equal proportions, and adjusted to a pH of 7 using NaOH (1 M). The resulting solution (40 mL) and 10 mL of the PCCA solution (0.2 g of PCCA dissolved in a pH 3.0 citric acid sodium citrate buffer) were mixed and stirred magnetically (85-2; Changzhou Guohua Electric Appliance Co., Ltd., Changzhou, China) for 2 h, and subsequently adjusted to a pH of 3.5 using hydrochloric acid (1 M). After centrifugation (8000× *g*, 30 min), the sediment was collected and freeze-dried to obtain a complex.

CS−PC−PCCA: the CS solution (1 mg/mL) was prepared with 1% acetic acid aqueous solution, followed by an ultrasonic treatment for 30 min and magnetic stirring at room temperature for 24 h. Thereafter, 10 mL of PCCA solution (0.3 g of PCCA dissolved in a pH 3.0 citric acid buffer) was mixed with 100 mL of CS solution and stirred magnetically for 40 min. Afterwards, 20 mL of PC solution (5 mg/mL, dissolved in deionized water) was slowly added to the solution above, and the pH was adjusted to 4.0 using NaOH (1 M) and homogenized (T25; Guangzhou Yike Laboratory Technology Co., Ltd., Guangzhou, China) for 5 min. After centrifuging (8000× *g*, 30 min), the sediment was collected and freeze-dried to obtain a complex.

### 2.3. Encapsulation Efficiency (EE)

As described in Section 2.3, the pH differential method [19] was used on the supernatant obtained after centrifugation to determine the PCCA content using an ultraviolet (UV) spectrophotometer (TU-1810; Beijing Puxie General Instrument Co., Ltd., Beijing, China) at 520 nm and 700 nm, and substituted into the following equation to calculate the EE [20]:

(1) $$\mathrm{EE}/\%=\frac{W_{0}-W_{1}}{W_{0}}\times 100$$

where *W*_0_ is the total concentration of PCCA (mg/mL) and *W*_1_ is the concentration of PCCA in the supernatant (mg/mL).

### 2.4. Physical Properties of the PCCA Complexes

#### 2.4.1. Determination of Solubility

With a slight modification to the method by Shittu & Lawal [21], 0.1 g of the complex was put into 10 mL of distilled water (30 °C) and stirred magnetically for 30 min. The suspension was centrifuged at 8000× *g* for 10 min, then the supernatant was collected and dried to a constant weight at 105 °C. The solubility is expressed as the percentage mass of the solid powder before and after drying.

#### 2.4.2. Determination of Hygroscopicity

With slight modifications to the method by Kanha et al. [22], the complex (1 g) was stored at 25 °C in a container containing saturated potassium chloride solution. After 7 days, the complex was removed and weighed. The hygroscopicity is the mass of the water absorbed per 100 g of the powder.

#### 2.4.3. Determination of Zeta Potential

The PCCA complex was dissolved in distilled water to a concentration of 1 mg/mL, and the zeta potential was measured using a ZS90 nanoparticle size and zeta potential analyzer (Malvern Instruments Ltd., Malvern, UK).

### 2.5. Structural Characterization of the PCCA Complexes

#### 2.5.1. Scanning Electron Microscopy (SEM)

Using a Hitachi Regulus 8100 scanning electron microscope (Hitachi Scientific Instruments Co., Ltd., Tokyo, Japan), the microstructures of the complexes were observed. The sample powder was placed on the stage, sprayed with gold using an MC1000 ion sputtering instrument, and observed with 10 k × power mirrors and 100 k × power mirrors.

#### 2.5.2. FTIR Analysis

Using a Fourier transform infrared spectrometer (VETEX8; Bruker, Karlsruhe, Germany), the infrared spectrum of the complex was scanned in the 4000−400 cm^−1^ range using the average true range (ATR) method.

#### 2.5.3. XRD Analysis

The complex was analysed using XRD (D8; Bruker, Karlsruhe, Germany) in the step scanning mode. The scan rate was 4°/min, the range was 5−60° (2θ), and the step width was 0.02°.

### 2.6. α-Glucosidase Inhibitory Ability

The inhibitory ability of PCCA on α-glucosidase was evaluated according to the description of Kumar et al. [23]. The absorbance was measured at 405 nm using a microplate reader (ELX-800; BioTek, Vermont, USA).

### 2.7. Simulated In Vitro Digestion of PCCA Complexes

An in vitro digestion was carried out with a slight modification of the description by Zang et al. [8]. Totals of 32.5 mg α-amylase, 1.2 g pepsin, 8 g trypsin, and 4.8 g bile salt, were dissolved in 25 mL of 1 mM CaCl_2_ (pH = 7), 30 mL of 0.1 M HCl solution, and 40 mL 0.1M NaHCO_3_, respectively, to simulate the saliva, gastric juice, and intestinal fluid.

Simulate mouth digestion: the sample (PCCA or the complex) was placed in a 20 mL NaCl solution (9 g/L) and mixed with 1 mL of simulated saliva at 37 °C, and shaken for 10 min. Simulate gastric digestion: the pH value was adjusted to 3.0, mixed with 2.5 mL of simulated gastric juice, and shaken for 2 h. Simulate intestinal digestion: the pH value was adjusted to 7.5, mixed with 5 mL of simulated intestinal fluid, and shaken for 2 h.

The digestive juice for each part was freeze-dried, dissolved in 70% ethanol solution, and centrifuged. The resulting supernatant was used to determine the concentration of the total and individual anthocyanins.

### 2.8. Determination of PCCA during Digestion Using High Performance Liquid Chromatography-Quadrupole Time of Flight Mass Spectrometer (HPLC-Q-TOF-MS)

Anthocyanins were identified and determined using HPLC-Q-TOF-MS systems (Agilent 1260 and Agilent 6530) equipped with an Agilent ORBAX SB-C18 column (4.6 × 100 mm, 1.8 μm). The column temperature was set to 30 °C and the injected sample volume was 5 μL. The mobile phase was composed of 0.1% formic acid in water (A) and acetonitrile (B) at a flow rate of 0.5 mL/min. The Gradient elution program was: (1) 0−55 min, 95−0% A; (2) 55−60 min, 0% A. The MS was equipped with an electrospray ionization (ESI) source and operated in a positive ion scanning mode (scan range: 100−1000 Da). The parameters for the MS identification were the gas temperature and flow with the sheath gas temperature and flow, which were 360 °C, 7 L/min, 400 °C, and 11 L/min, respectively; fragmentor of 150 V; collision energy of 20/30/40 V. The MS parameters for the quantification were the gas temperature and flow with the sheath gas temperature and flow which were 350 °C, 8 L/min, 380 °C, and 11 L/min, respectively. The anthocyanin quantification was carried out using an external standard method. Cyanidin-, peonidin-, pelargonidin-, malvidin, petunidin-3-O-glucoside were used to make the standard curve and their equivalents were used to express the content of their derivatives. The standard curves were as follows: y = 602.7x + 34,288 (R^2^ = 0.9993), y = 188.93x + 86,944 (R^2^ = 0.9992), y = 109.5x + 17,196 (R^2^ = 0.9995), y = 156.37x + 23,146 (R^2^ = 0.9992), and y = 1818.3x + 1,000,000 (R^2^ = 0.9995). The linear range was 0.01−1000 (μg/mL).

### 2.9. Statistical Analysis

All the experiments were repeated in triplicate. The results are expressed as the mean ± standard deviation (SD). The statistical analysis (*p* < 0.05) was done using SPSS 26 software.

## 3. Results and Discussion

### 3.1. Encapsulation Efficiency and Physical Properties of the PCCA Complexes

Table 1 shows that the EEs for WPI−PC−PCCA and CS−PC−PCCA are higher than those for PC−PCCA and WPI−PCCA (*p* < 0.05), indicating that the combined material was more efficient for the encapsulation of PCCA. A low solubility can help delay the release of complexes, mainly due to a complicated coagulation [24]. The solubility of the 4 complexes in the high to low order was WPI−PC−PCCA > WPI−PCCA > PC−PCCA > CS−PC−PCCA (*p* < 0.05), indicating that CS−PC−PCCA has the best-sustained release among the four complexes.

The hygroscopic values for the four complexes ranged from 10.23 ± 0.28 (CS−PC−PCCA) to 20.93 ± 0.12 (WPI−PCCA) g/100 g, with significant differences (*p* < 0.05). The high hygroscopicity of the composite leads to agglomeration, reducing the retention of the active components and hindering their dispersion [25]. In contrast, a low hygroscopic complex is in a glassy state, which forms a protective barrier to reduce the influence of external factors such as oxidation [26].

The greater the absolute value of the zeta potential, the better the stability, while the smaller the potential, the easier it is for the molecules to agglomerate [27]. The absolute values of the zeta potential for WPI−PCCA and WPI−PC−PCCA were significantly lower than that for CS−PC−PCCA (*p* < 0.05) but significantly higher than that for PC−PCCA (*p* < 0.05), suggesting that the stability of the CS−PC−PCCA solution was the best, while that of PC−PCCA was the worst.

### 3.2. Structural Characterization of the Steady-State PCCA Complex

#### 3.2.1. SEM Analysis

**Figure 1:**
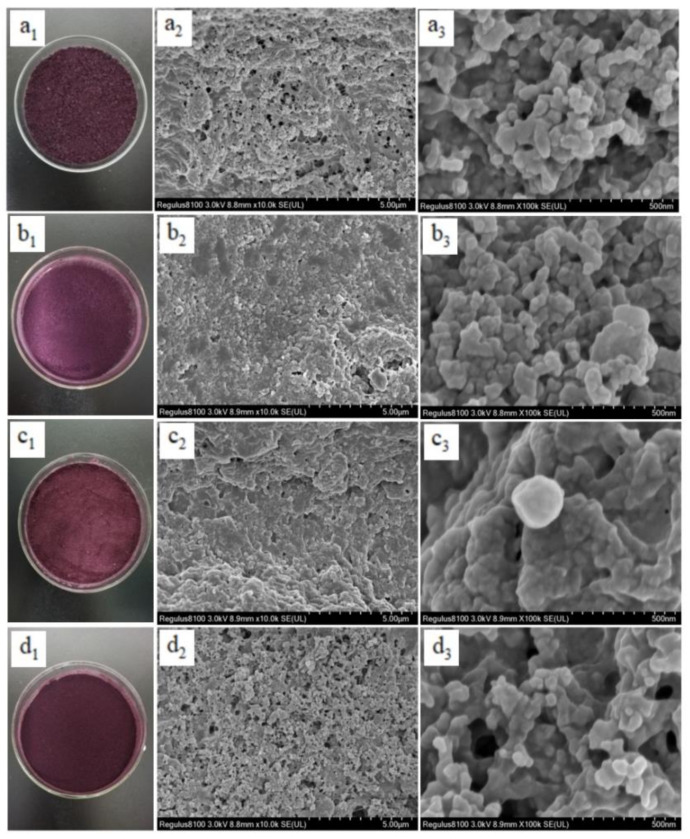
Scanning electron microscopy (SEM) images of PC−PCCA, WPI−PCCA, WPI−PC−PCCA, and CS−PC−PCCA. (**a_1_**−**d_1_**) are the morphologies observed under the digital camera; (**a_2_**−**d_2_**) are the morphologies observed under the SEM at 10 k × multiples; and (**a_3_**−**d_3_**) are the morphologies observed under the SEM at 100 k × multiples.

The apparent structure of the complex after freeze-drying was observed using a scanning electron microscope. Figure 1 shows that PC−PCCA, WPI−PCCA, and WPI−PC−PCCA are spherical or oval and aggregate easily. The particle sizes are 100−200, 200−300, and 300−400 nm, respectively. CS−PC−PCCA is multiparticulate and irregular in shape with particle sizes in the 200−300 nm range, making aggregation easy (Figure 1d_2_) [10]. Moreover, smaller secondary particles are also found at 100 × K times (Figure 1d_3_), which may be caused by the surface shrinkage and automatic aggregation of PCCA, CS, and PC during the freeze-drying [10]. In addition, at 100 × K times, the surfaces of the four complexes are rough due to a partial PCCA adsorption onto the surface of the complex. Additionally, freeze-drying promotes the disintegration of the aggregates through the fracture of the electrostatic bonds, resulting in an aggregated grid structure [26].

#### 3.2.2. FTIR Analysis

PCCA has strong absorptions at 3268, 2977, 1723, and 1474 cm^−1^, which are due to the influence of O−H and C−H bond stretching vibrations, benzopyran ring (C=O), and C=C vibration [7,28]. In addition, PCCA had C−O vibration peaks at 1214 and 1039 cm^−1^ and a C−H bending vibration peak at 844 cm^−1^ [28]. The four complexes had a characteristic PCCA absorption peak, but the intensity was weakened due to the interaction of PCCA with the encapsulation material.

**Figure 2:**
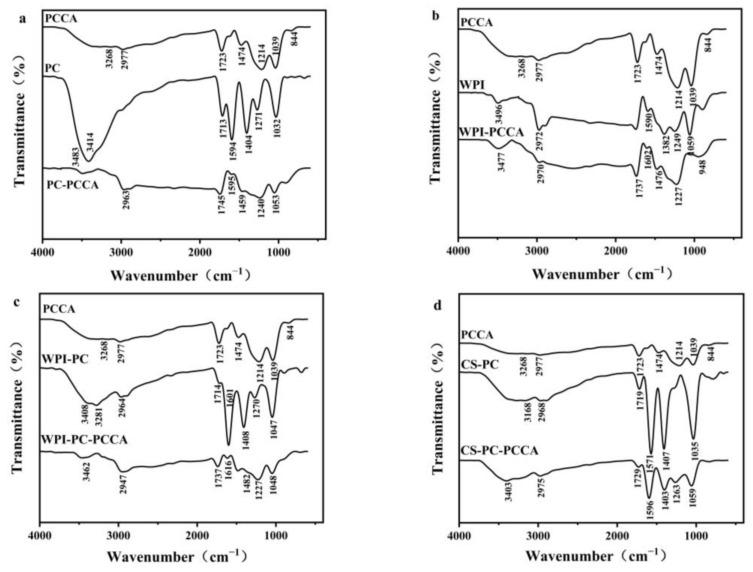
Fourier transform infrared spectroscopy (FTIR) spectra of PC−PCCA, WPI−PCCA, WPI−PC−PCCA, and CS−PC−PCCA (**a**−**d**).

Figure 2a shows that there is an O−H stretching vibration peak at 3414 cm^−1^ for PC, and a C=O vibration absorption peak in the 1713−1594 cm^−1^ range [29]. In addition, the absorption peak of PC at 1404 cm^−1^ is caused by the symmetrical stretching vibration of the carboxylic acid functional groups, whereas the peaks near 1271 cm^−1^ and 1032 cm^−1^ are caused by the stretching vibration of the C−O bond in carboxylic acid [30]. After adding PCCA, an O−H stretching vibration peak at 3483 cm^−1^ is observed, indicating that there is a hydrogen bond in PC−PCCA [31]. The absorption peaks at 1594, 1404, 1271, and 1032 cm^−1^ shifted slightly and are significantly weakened, indicating that PC and PCCA are joined by hydrogen bonds [31].

Figure 2b,c shows that WPI and WPI−PC have wide peaks near 3400 cm^−1^ and after adding PCCA, the peak widens and shifts slightly, which may be due to the stretching vibration of O−H and intermolecular hydrogen bonds, indicating that there is a hydrogen bond between PCCA and WPI in WPI−PC [32]. Figure 2b shows that after WPI combines with PCCA, the amide I band shifts from 1590 cm^−1^ to 1602 cm^−1^ (affected by the C=O tensile vibration), meaning that the α-helix content in WPI and the strength of the amide I band decreases. This indicates the interaction of the hydrogen bonds and the hydrophobic attraction between WPI and PCCA [8].

Figure 2d shows that the absorption band of CS−PC−PCCA near 3403 cm^−1^ is generated by the tensile vibration of the O−H and N−H groups. Compared with CS−PC, the absorption band shifts, indicating that CS−PC and PCCA are joined by hydrogen bonds [33]. In addition, CS−PC−PCCA also corresponds to the C=O, N−H, and C−N stretching vibration peak of the amide group near the wavelengths of 1729, 1596, and 1403 cm^−1^, respectively [34]. However, compared with CS−PC, the absorption bands of CS−PC−PCCA at 1729, 1596, and 1403 cm^−1^ are slightly wider with significantly decreased intensities, indicating that the amino group in CS−PC has intermolecular electrostatic interactions with PCCA [29].

#### 3.2.3. XRD Analysis

**Figure 3:**
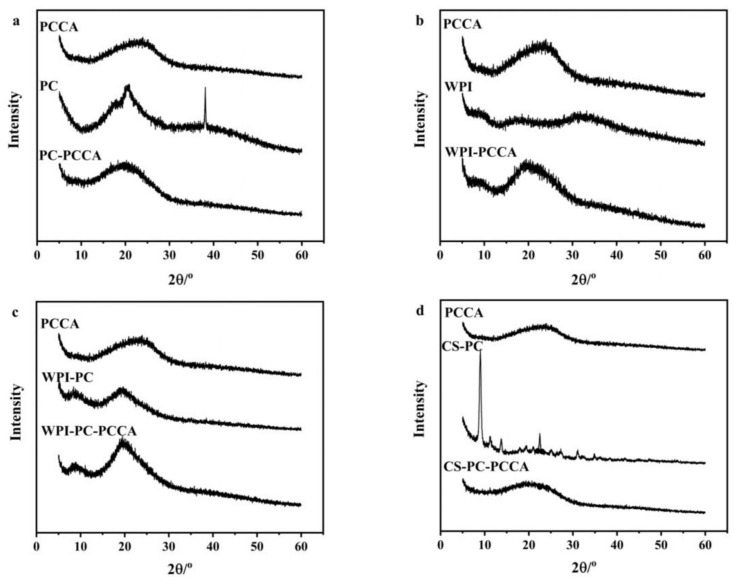
X-ray diffraction (XRD) spectra of PC−PCCA, WPI−PCCA, WPI−PC−PCCA, and CS−PC−PCCA (**a**−**d**).

Crystallinity can be determined from the diffraction peaks in the spectrum. Sharp and narrow diffraction peaks indicate a high crystallinity, whereas wide diffraction peaks indicate an amorphous state [35]. Figure 3 shows that PCCA has a wide, strong diffraction peak at 2θ = 23.61°, indicating the existence of an amorphous region, which is the same as the XRD diffraction peak shape of blueberry anthocyanins reported by Liu et al. [36]. Figure 3a shows that PC is amorphous due to its wide diffraction peak. However, there are relatively sharp characteristic diffraction peaks near 2θ = 17.62° and 20.47°, indicating the existence of a crystalline part [37]. PC−PCCA has a wide diffraction peak at 2θ = 19.11°, and the crystallization peaks of PC at 17.62° and 20.47° disappeared, indicating that PC and PCCA combined and exist in an amorphous form.

Figure 3b shows that WPI has dispersion diffraction peaks at 2θ = 8.42°, 17.12°, and 30.43°, indicating that it has semicrystalline characteristics with a low crystallinity [38]. However, WPI−PCCA has a weak diffraction peak at 2θ = 9.68° and a wide and strong diffraction peak at 2θ = 19.53°, indicating that WPI−PCCA is amorphous. As shown in Figure 3c the types of diffraction peaks for WPI−PC and WPI−PC−PCCA are similar, both are wide and strong, indicating their existence in amorphous forms. However, the WPI−PC−PCCA diffraction peak near 20.29 ° is sharper and higher than that of WPI−PC (19.38°), indicating that the WPI−PC and PCCA combination improves the crystallinity [35].

Figure 3d shows that the diffraction peaks of CS−PC at 2θ = 9.01°, 11.14°, 13.71°, and 22.49° show that it has semicrystalline characteristics, and the peaks at 9.01° and 11.14° may be related to the crystal structure of CS [29,39]. CS−PC−PCCA has a wider diffraction peak than PCCA at 2θ = 19.25°, and the CS−PC crystallization peak disappeared. It may be because of the intermolecular interaction between CS−PC and PCCA, which limits the molecular movement and forms a more amorphous CS−PC−PCCA [39,40].

### 3.3. α-Glucosidase Inhibition Ability

**Figure 4:**
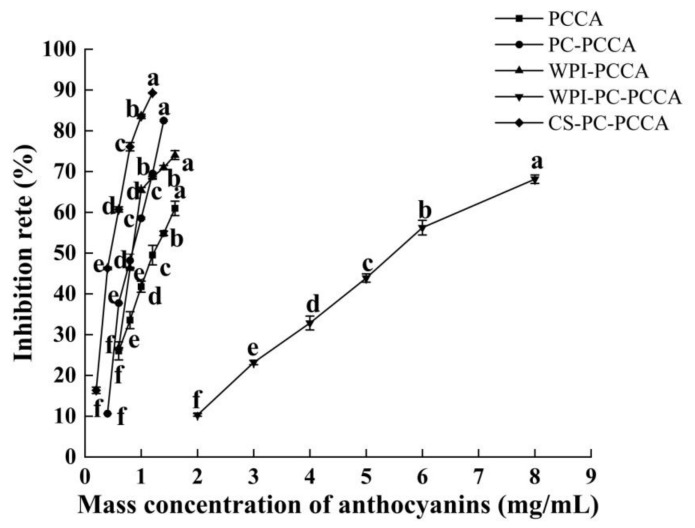
α-glucosidase inhibition activity. Error bars mark the standard error. a−f indicates significant difference (*p* < 0.05).

The α-glucosidase inhibition rates of PCCA, PC−PCCA, WPI−PCCA, WPI−PC−PCCA, and CS−PC−PCCA significantly increased with the increasing concentrations (Figure 4). Their IC_50_ values were 1.221 ± 0.141, 0.815 ± 0.006, 0.869 ± 0.001, 5.504 ± 0.105, and 0.45 ± 0.002 mg/mL, respectively, indicating that combining PC, WPI, and CS−PC with PCCA can increase the hypoglycaemic activity of PCCA. Studies have shown that anthocyanins can bind to the active site of α-glucosidase and inhibit its catalytic activity through hydrogen bonding to exert a hypoglycaemic effect. The binding energy and the number of binding sites were the main factors affecting the inhibitory ability of α-glucosidase [41,42].

### 3.4. Simulated In Vitro Digestion

#### 3.4.1. Stability of Total Anthocyanins Content (TAC) during Digestion of PCCA and Its Complexes

Table 2 shows that the TAC decreased significantly during the stimulated digestions, especially after it simulated an intestinal digestion for 2 h. The PCCA retention rate is 13.09 ± 0.05%, while the retention rates for PC−PCCA, WPI−PCCA, WPI−PC−PCCA, and CS−PC−PCCA are 23.84 ± 0.12%, 22.77 ± 0.18%, 19.47 ± 0.07%, and 25.18 ± 0.28%, respectively, indicating that the complexes significantly improve the stability of PCCA in the intestinal digestion stage and has a protective effect on PCCA, among which CS−PC−PCCA has the best effect. After the simulated mouth digestion, the degradation loss of PCCA was small, which may be due to the inhibition of α-amylase by PCCA and the short digestion time. When the simulated stomach digestion lasted for 1 h, the retention rates of WPI−PCCA and WPI−PC−PCCA were 90.13 ± 0.19% and 88.96 ± 0.12%, respectively, being slightly lower than that for PCCA 90.27 ± 0.62%, which may be due to the denaturation and dissociation of the protein molecules under the influence of acidic gastric juice, resulting in the damage of the anthocyanin content [17].

#### 3.4.2. Stability of Individual Anthocyanins during Digestion of PCCA and Its Complexes

Twenty-three individual anthocyanins were identified from PCCA (Table 3). Based on the stability of TAC, PCCA, PC−PCCA, WPI−PCCA, and CS−PC−PCCA with good protective PCCA abilities were selected to a further study of the stability of individual anthocyanins during gastrointestinal digestion (Table 4). Two hours after gastric digestion, the cyanidin, cyanidin-3-O-sambubioside, malvidin, malvidin-3-O-rutinoside, peonidin-3,5-diglucoside, peonidin-3-O-(dimalonylglucoside), peonidin-3-O-(6″-ethyl malonylglucoside), and petunidin-3-rutinoside were slightly higher than those in the original state. Their content increased by 1.80−28.99%. The increase in the contents of cyanidin and malvidin may be because the intermediate products produced by the disaccharide reaction of cyanidin-3-O-glucoside and malvidin-3-O-glucoside had not been further oxidized to other substances [43]. The increase in the diglucoside content may be due to the glycosyl substitution at the C_5_ position, which reduces the nucleophilicity of the C_6_ and C_8_ positions, making PCCA less vulnerable to an electrophilic attack [44]. The temporary increase in the content of other monomers may be caused by the transformation of anthocyanins under the influence of digestive enzymes and other external factors (such as the acidic environment in the stomach) [17,45]. After an intestinal digestion for 2 h, the individual anthocyanin contents decreased significantly. Cyanidin-3-O-glucoside, petunidin, cyanidin, petunidin-3-rutinoside, cyanidin-3-O-sambubioside, and peonidin-3,5-diglucoside were not detected. This is mainly because the alkaline environment of intestinal fluid leads to the degradation of anthocyanins, making them unstable in the intestine [46]. However, the retention rates for the other detected monomers were higher during the digestion of the complexes than during that of PCCA, showing that the complexes have good PCCA protective effects

## 4. Conclusions

Four types of PCCA complexes, PC−PCCA, WPI−PCCA, WPI−PC−PCCA, and CS−PC−PCCA, were prepared. The four complexes exhibited different shapes and sizes (100−400 nm), encapsulation efficiencies (41.80 ± 0.05−58.84 ± 2.82%), physical properties, crystallinity, and combination modes through hydrogen bonding, a hydrophobic interaction, and an electrostatic interaction with PCCA, thus affecting their protective ability. In general, PC−PCCA, WPI−PCCA, and CS−PC−PCCA significantly improved the inhibition of α-glucosidase, the total PCCA content, and the individual anthocyanins in the simulated digestion process. Among them, CS−PC−PCCA is multiparticulate and irregular in shape, owing to the combination of electrostatic interactions and hydrogen bonds. Moreover, CS−PC−PCCA (48.13 ± 2.73%) has an EE second only to WPI−PC−PCCA, a medium size (200−300 nm), the weakest hygroscopicity (10.23 ± 0.28), the lowest solubility (10.57 ± 1.26), and the highest zeta potential (28.20 ± 1.14). In addition, it exhibited the best α-glucosidase inhibition and simulated digestion stability. Therefore, loading PCCA into a complex containing CS and PC is of a great significance in stabilizing PCCA and improving its hypoglycaemic ability, which helps expand the application of PCCA.

## Figures and Tables

**Table 1:** Encapsulation yield and physical properties of steady-state complexes from PCCA.

	EE/%	Solubility/%	Hygroscopicity/%	ζ-Potential/mV
PC−PCCA	45.83% ± 0.45 ^c^	12.30 ± 0.98 ^c^	13.10 ± 0.45 ^c^	7.18 ± 0.61 ^c^
WPI−PCCA	41.80% ± 0.05 ^d^	20.73 ± 0.25 ^b^	20.93 ± 0.12 ^a^	−13.21 ± 0.54 ^b^
WPI−PC−PCCA	58.84% ± 2.82 ^a^	28.16 ± 1.38 ^a^	13.93 ± 0.19 ^b^	8.04 ± 0.26 ^c^
CS−PC−PCCA	48.13% ± 2.73 ^b^	10.57 ± 1.26 ^c^	10.23 ± 0.28 ^d^	28.20 ± 1.14 ^a^

Note: Different lowercase letters in the same column indicate significant difference (*p* < 0.05).

**Table 2:** The changes in TAC during in vitro digestion of PCCA and its four complex.

	Total Anthocyanin Content (mg/L)					
	Origin	Mouth	Gastric 1 h	Gastric 2 h	Intestinal 1 h	Intestinal 2 h
PCCA	580.36 ± 4.82 ^a^	550.61 ± 3.16 ^b^	523.56 ± 2.89 ^c^	491.48 ± 3.21 ^d^	127.64 ± 2.21 ^e^	75.84 ± 0.98 ^f^
PC−PCCA	395.64 ± 1.77 ^a^	387.25 ± 2.14 ^b^	369.62 ± 4.14 ^c^	363.51 ± 2.44 ^d^	162.33 ± 1.36 ^e^	94.32 ± 1.16 ^f^
WPI−PCCA	481.37 ± 2.28 ^a^	467.49 ± 2.49 ^b^	438.08 ± 2.36 ^c^	419.90 ± 2.67 ^d^	179.87 ± 1.44 ^e^	109.61 ± 1.27 ^f^
WPI−PC−PCCA	413.39 ± 3.27 ^a^	394.38 ± 3.27 ^b^	367.47 ± 1.19 ^c^	351.44 ± 1.03 ^d^	138.88 ± 0.93 ^e^	80.39 ± 1.08 ^f^
CS−PC−PCCA	405.05 ± 2.14 ^a^	396.22 ± 2.55 ^b^	387.87 ± 2.16 ^c^	374.47 ± 2.14 ^d^	159.02 ± 2.48 ^e^	101.26 ± 3.42 ^f^

Note: Origin represents the content of total anthocyanins in the complex before digestion; different lowercase letters in the same row of data indicate significant differences (*p* < 0.05).

**Table 3:** Results of mass spectra identification of individual anthocyanins.

NO. (Peak)	t_R_ (min)	Precursor Ion (*m*/*z*)	Theoretical Mass (*m*/*z*)	Proposed Formula	Product Ions (*m*/*z*)	Proposed Structure
1	9.210	287.0552	287.055	C_15_H_11_O_6_	213.0534	Cyanidin
2	10.136	621.1091	621.1086	C_27_H_25_O_17_	287.0555	Cyanidin-3-O-(3″,6″-dimalonylglucoside)
3	9.260	535.1084	535.1082	C_24_H_22_O_14_	287.0540	Cyanidin-3-O-(6″-malonylglucoside)
4	8.318	449.1092	449.1078	C_21_H_21_O_11_	287.0557	Cyanidin-3-O-glucoside
5	10.253	577.1238	577.1188	C_26_H_25_O_15_	287.0600	Cyanidin-3-O-sambubioside
6	11.802	331.0820	331.0812	C_17_H_15_O_7_	315.0498	Malvidin
7	11.751	493.1343	493.1346	C_23_H_25_O_12_	331.0801	Malvidin-3-O-glucoside
8	12.610	579.1360	579.1344	C_26_H_27_O_15_	331.0798	Malvidin-3-O-(6″-malonylglucoside)
9	12.088	639.1613	639.1556	C_28_H_31_O_17_	331.0811	Malvidin-3-O-rutinoside
10	8.637	271.0647	271.0606	C_15_H_11_O_5_	253.0537	Pelargonidin
11	5.911	595.1658	595.1657	C_27_H_31_O_15_	271.0592	Pelargonidin-3,5-diglucoside
12	8.722	433.1134	433.1129	C_21_H_21_O_10_	271.0603	Pelargonidin-3-O-glucoside
13	9.732	519.1140	519.1133	C_24_H_23_O_13_	271.0602	Pelargonidin-3-O-(6″-malonylglucoside)
14	10.455	605.1167	605.1137	C_27_H_25_O_16_	271.0593	Pelargonidin-3-O-(dimalonylglucoside)
15	7.510	625.1782	625.1763	C_28_H_33_O_16_	301.0706	Peonidin-3,5-diglucoside
16	10.321	549.1253	549.1239	C_25_H_25_O_14_	301.0707	Peonidin-3-O-(6″-malonylglucoside)
17	12.004	577.1569	577.1552	C_27_H_29_O_14_	301.0719	Peonidin-3-O-(6″-ethyl malonylglucoside)
18	10.489	635.1247	635.1243	C_28_H_27_O_17_	301.0710	Peonidin-3-O-(dimalonylglucoside)
19	8.907	463.1230	463.1240	C_22_H_23_O_11_	301.0695	Peonidin-3-O-glucoside
20	11.196	591.1383	591.1344	C_27_H_27_O_15_	301.0694	Peonidin-3-O-sambubioside
21	11.903	317.0676	317.0656	C_16_H_13_O_7_	302.0446	Petunidin
22	11.635	625.1765	625.1763	C_28_H_33_O_16_	317.0647	Petunidin-3-rutinoside
23	12.913	565.1200	565.1188	C_25_H_24_O_15_	317.0648	Petunidin-3-O-(6″-malonylglucoside)

**Table 4:** Changes in anthocyanin monomers content during in vitro digestion of steady-state complex.

NO. (Peak)	Proposed Structure	PCCA			PC−PCCA			WPI−PCCA			CS−PC−PCCA		
		Origin	Gastric 2 h	Intestinal 2 h	Origin	Gastric 2 h	Intestinal 2 h	Origin	Gastric 2 h	Intestinal 2 h	Origin	Gastric 2 h	Intestinal 2 h
1	Cyanidin	0.27 ± 0.03 ^a^	0.29 ± 0.01 ^a^	ND	0.09 ± 0.01 ^a^	0.10 ± 0.03 ^a^	ND	0.10 ± 0.02 ^a^	0.09 ± 0.01 ^a^	ND	0.05 ± 0.01 ^a^	0.06 ± 0.00 ^a^	0.01 ± 0.00 ^b^
2	Cyanidin-3-O-(3″,6″-dimalonylglucoside)	1.32 ± 0.08 ^a^	0.97 ± 0.05 ^b^	0.25 ± 0.08 ^c^	0.75 ± 0.06 ^a^	0.49 ± 0.04 ^b^	0.15 ± 0.05 ^c^	1.16 ± 0.08 ^a^	0.69 ± 0.03 ^b^	0.20 ± 0.02 ^c^	1.09 ± 0.22 ^a^	0.68 ± 0.08 ^b^	0.11 ± 0.06 ^c^
3	Cyanidin-3-O-(6″-malonylglucoside)	0.34 ± 0.05 ^a^	0.25 ± 0.04 ^b^	0.08 ± 0.02 ^c^	0.23 ± 0.04 ^a^	0.15 ± 0.04 ^b^	0.06 ± 0.07 ^c^	0.21 ± 0.04 ^a^	0.11 ± 0.01 ^b^	0.04 ± 0.05 ^c^	0.16 ± 0.01 ^a^	0.13 ± 0.02 ^b^	0.05 ± 0.02 ^c^
4	Cyanidin-3-O-glucoside	74.23 ± 0.81 ^a^	53.35 ± 0.43 ^b^	ND	43.91 ± 0.07 ^a^	25.31 ± 0.21 ^b^	ND	91.30 ± 0.20 ^a^	64.57 ± 0.03 ^b^	ND	95.14 ± 0.30 ^a^	69.64 ± 0.13 ^b^	ND
5	Cyanidin-3-O-sambubioside	7.83 ± 0.51 ^a^	6.84 ± 0.16 ^b^	ND	20.96 ± 0.42 ^b^	24.46 ± 0.46 ^a^	ND	20.94 ± 0.66 ^a^	19.44 ± 0.34 ^b^	0.33 ± 0.02 ^c^	20.83 ± 0.85 ^b^	22.65 ± 0.49 ^a^	ND
6	Malvidin	23.98 ± 0.14 ^c^	16.21 ± 0.12 ^b^	4.63 ± 0.34 ^c^	63.83 ± 0.49 ^b^	71.78 ± 0.73 ^a^	14.11 ± 0.16 ^c^	84.61 ± 0.87 ^b^	100.73 ± 0.2 ^a^	17.50 ± 0.07 ^c^	57.48 ± 0.04 ^b^	71.33 ± 0.43 ^a^	15.93 ± 0.34 ^c^
7	Malvidin-3-O-glucoside	5.46 ± 0.02 ^a^	1.89 ± 0.21 ^b^	0.17 ± 0.01 ^c^	0.36 ± 0.03 ^a^	0.21 ± 0.01 ^b^	0.02 ± 0.02 ^c^	1.06 ± 0.08 ^a^	0.67 ± 0.01 ^b^	0.07 ± 0.11 ^c^	1.24 ± 0.31 ^a^	0.87 ± 0.26 ^b^	0.12 ± 0.03 ^c^
8	Malvidin-3-O-(6″-malonylglucoside)	8.02 ± 0.25 ^a^	4.72 ± 0.23 ^b^	0.11 ± 0.02 ^c^	7.39 ± 0.33 ^a^	2.63 ± 0.12 ^b^	1.17 ± 0.22 ^c^	3.66 ± 0.44 ^a^	2.54 ± 0.13 ^b^	0.92 ± 0.24 ^c^	5.91 ± 0.41 ^a^	2.68 ± 0.46 ^b^	0.15 ± 0.04 ^c^
9	Malvidin-3-O-rutinoside	1.78 ± 0.02 ^a^	1.37 ± 0.10 ^a^	0.43 ± 0.41 ^b^	1.69 ± 0.04 ^b^	2.18 ± 0.91 ^a^	0.52 ± 0.07 ^c^	2.54 ± 0.23 ^a^	1.67 ± 0.91 ^ab^	0.68 ± 0.02 ^b^	1.85 ± 0.08 ^b^	2.32 ± 0.12 ^a^	0.59 ± 0.04 ^c^
10	Pelargonidin	26.66 ± 0.86 ^a^	21.55 ± 0.44 ^b^	0.21 ± 0.03 ^c^	5.52 ± 0.29 ^a^	4.53 ± 0.03 ^b^	0.39 ± 0.02 ^b^	1.98 ± 0.06 ^a^	1.16 ± 0.03 ^b^	0.19 ± 0.03 ^c^	1.88 ± 0.02 ^a^	1.51 ± 0.02 ^b^	0.21 ± 0.00 ^c^
11	Pelargonidin-3,5-diglucoside	2.15 ± 0.03 ^a^	1.92 ± 0.23 ^a^	0.59 ± 0.08 ^b^	3.60 ± 0.05 ^a^	2.64 ± 0.05 ^b^	1.02 ± 0.15 ^c^	3.18 ± 0.42 ^a^	2.53 ± 0.16 ^b^	0.92 ± 0.07 ^c^	2.73 ± 0.08 ^a^	2.38 ± 0.19 ^a^	0.87 ± 0.25 ^b^
12	Pelargonidin-3-O-glucoside	210.31 ± 0.8 ^a^	158.82 ± 0.2 ^b^	20.53 ± 0.62 ^c^	66.74 ± 0.67 ^a^	44.68 ± 0.61 ^b^	18.07 ± 0.30 ^c^	79.67 ± 0.53 ^a^	52.6 ± 0.72 ^b^	16.49 ± 0.41 ^c^	57.73 ± 0.47 ^a^	40.66 ± 0.21 ^b^	16.92 ± 0.06 ^c^
13	Pelargonidin-3-O-(6″-malonylglucoside)	107.30 ± 0.6 ^a^	98.40 ± 0.51 ^b^	7.29 ± 0.15 ^c^	74.71 ± 0.19 ^a^	69.74 ± 0.34 ^b^	7.09 ± 0.23 ^c^	38.13 ± 2.32 ^a^	32.20 ± 0.70 ^b^	3.98 ± 0.68 ^c^	66.61 ± 0.33 ^a^	61.87 ± 0.91 ^b^	12.62 ± 0.65 ^c^
14	Pelargonidin-3-O-(dimalonylglucoside)	4.09 ± 0.55 ^a^	3.61 ± 0.08 ^b^	0.82 ± 0.09 ^c^	9.96 ± 0.44 ^a^	9.02 ± 0.57 ^b^	2.01 ± 0.22 ^c^	5.25 ± 0.19 ^a^	4.93 ± 0.47 ^a^	1.27 ± 0.61 ^b^	3.00 ± 0.62 ^a^	2.79 ± 0.49 ^a^	0.78 ± 0.21 ^a^
15	Peonidin-3,5-diglucoside	1.22 ± 0.04 ^a^	1.04 ± 0.01 ^b^	ND	3.77 ± 0.21 ^b^	4.18 ± 0.29 ^a^	0.26 ± 0.02 ^c^	7.07 ± 0.30 ^b^	8.14 ± 0.59 ^a^	ND	5.94 ± 0.18 ^b^	6.97 ± 0.03 ^a^	ND
16	Peonidin-3-O-(6″-malonylglucoside)	1.87 ± 0.03 ^a^	1.60 ± 0.07 ^b^	0.04 ± 0.02 ^c^	10.21 ± 0.21 ^a^	9.30 ± 0.22 ^b^	1.01 ± 0.01 ^c^	5.42 ± 0.07 ^a^	4.41 ± 0.03 ^b^	ND	17.89 ± 0.03 ^a^	15.70 ± 0.24 ^b^	1.83 ± 0.04 ^c^
17	Peonidin-3-O-(6″-ethyl malonylglucoside)	24.71 ± 0.43 ^b^	26.44 ± 0.70 ^a^	0.24 ± 0.05 ^c^	66.58 ± 0.50 ^b^	76.14 ± 0.72 ^a^	ND	66.52 ± 7.03 ^a^	67.72 ± 0.77 ^a^	0.77 ± 0.08 ^b^	66.17 ± 0.50 ^b^	72.96 ± 0.73 ^a^	1.27 ± 0.06 ^c^
18	Peonidin-3-O-(dimalonylglucoside)	5.52 ± 0.25 ^a^	4.80 ± 0.09 ^b^	ND	7.75 ± 0.47 ^b^	8.79 ± 0.62 ^a^	1.22 ± 0.06 ^c^	11.90 ± 0.91 ^b^	13.41 ± 0.62 ^a^	2.04 ± 0.09 ^c^	13.52 ± 0.82 ^b^	15.02 ± 0.80 ^a^	2.70 ± 0.04 ^c^
19	Peonidin-3-O-glucoside	68.04 ± 0.37 ^a^	61.49 ± 0.42 ^b^	18.09 ± 0.68 ^c^	20.54 ± 0.59 ^a^	19.04 ± 0.13 ^b^	6.09 ± 0.54 ^c^	21.82 ± 0.80 ^a^	20.30 ± 0.40 ^a^	6.14 ± 0.40 ^b^	23.14 ± 1.39 ^a^	21.71 ± 0.25 ^b^	7.15 ± 0.26 ^c^
20	Peonidin-3-O-sambubioside	5.38 ± 0.26 ^a^	4.51 ± 1.54 ^ab^	0.91 ± 0.86 ^b^	11.03 ± 2.74 ^a^	9.51 ± 1.57 ^ab^	2.78 ± 1.26 ^b^	11.72 ± 0.59 ^a^	10.38 ± 3.72 ^a^	2.61 ± 1.09 ^b^	4.79 ± 1.51 ^a^	4.31 ± 1.18 ^a^	1.13 ± 1.71 ^a^
21	Petunidin	3.09 ± 0.14 ^b^	2.89 ± 0.52 ^a^	ND	3.62 ± 0.23 ^a^	3.46 ± 0.04 ^b^	ND	2.94 ± 0.18 ^a^	2.79 ± 0.06 ^b^	0.02 ± 0.01 ^c^	1.09 ± 0.05 ^a^	0.98 ± 0.03 ^b^	ND
22	Petunidin-3-rutinoside	0.03 ± 0.00 ^a^	0.03 ± 0.01 ^a^	ND	0.38 ± 0.04 ^ab^	0.41 ± 0.46 ^a^	0.02 ± 0.00 ^b^	0.73 ± 0.02 ^b^	0.84 ± 0.05 ^a^	ND	0.61 ± 0.05 ^b^	0.72 ± 0.02 ^a^	ND
23	Petunidin-3-O-(6″-malonylglucoside)	0.67 ± 0.08 ^a^	0.62 ± 0.07 ^a^	0.19 ± 0.04 ^b^	21.53 ± 0.04 ^a^	19.49 ± 0.35 ^b^	6.49 ± 0.04 ^c^	30.23 ± 0.11 ^a^	26.97 ± 0.07 ^b^	8.484 ± 0.03 ^c^	0.67 ± 0.03 ^a^	0.59 ± 0.07 ^b^	0.20 ± 0.05 ^c^

Note: Values were expressed as μg/mL; all samples were subjected to simulated in vitro gastrointestinal digestion after simulated mouth digestion; origin represents the monomer content of anthocyanins in the pre digestion complex; different lowercase letters in the same row of data indicate that the content of anthocyanin monomer in PCCA, PC−PCCA, WPI−PCCA and CS−PC−PCCA at different stages is significantly different (*p* < 0.05); Nd indicates not detected.

## Data Availability

The data presented in this study are available on request from the corresponding author.
